# Ectopic Expression of Testis Germ Cell Proteins in Cancer and Its Potential Role in Genomic Instability

**DOI:** 10.3390/ijms17060890

**Published:** 2016-06-06

**Authors:** Aaraby Yoheswaran Nielsen, Morten Frier Gjerstorff

**Affiliations:** Department of Cancer and Inflammation Research, Institute for Molecular Medicine, University of Southern Denmark, Odense DK-5000, Denmark; aynielsen@health.sdu.dk

**Keywords:** genomic instability, cancer development, testis germ cell proteins, mitosis, polyploidy, mitotic fidelity

## Abstract

Genomic instability is a hallmark of human cancer and an enabling factor for the genetic alterations that drive cancer development. The processes involved in genomic instability resemble those of meiosis, where genetic material is interchanged between homologous chromosomes. In most types of human cancer, epigenetic changes, including hypomethylation of gene promoters, lead to the ectopic expression of a large number of proteins normally restricted to the germ cells of the testis. Due to the similarities between meiosis and genomic instability, it has been proposed that activation of meiotic programs may drive genomic instability in cancer cells. Some germ cell proteins with ectopic expression in cancer cells indeed seem to promote genomic instability, while others reduce polyploidy and maintain mitotic fidelity. Furthermore, oncogenic germ cell proteins may indirectly contribute to genomic instability through induction of replication stress, similar to classic oncogenes. Thus, current evidence suggests that testis germ cell proteins are implicated in cancer development by regulating genomic instability during tumorigenesis, and these proteins therefore represent promising targets for novel therapeutic strategies.

## 1. Introduction

In normal cells, genomic stability is maintained by multiple highly controlled mechanisms that secure fidelity of DNA replication during S phase, segregation of chromosomes during mitosis, and precise repair of DNA damage throughout the cell cycle [1]. Genomic instability, on the other hand, is a hallmark of almost all human cancers and is acknowledged as a main driving force of tumorigenesis [2]. Changes in the genome resulting from genomic instability include mutations, rearrangements of chromosomes, gain or loss of partial or whole chromosomes, *etc.* In most cases, a significant change to the genome results in a non-viable cell, but in rare events it might confer a selective advantage on a specific cell leading to cancer initiation or progression [2]. The development of high-throughput techniques for DNA sequencing has enabled large-scale analysis of cancer genomes to identify common and rare genomic alterations that may support tumorigenesis. As expected, such genomic changes often involve genes encoding tumor suppressors or proto-oncogenes. For instance, the *TP53* gene is deleted or mutated in more than 50% of human cancers, resulting in abolished function of the encoded p53 tumor suppressor [3]. Similarly, proto-oncogenes can be activated by mutations (e.g., KRAS [4], BRAF [4], EGFR [5]), focal amplification (e.g., HER2 [6], EGFR [7]) or genomic rearrangements creating fusion oncogenes (e.g., BCR-ABL [8], SYT-SSX [9]). Recent evidence further demonstrates that genomic alterations in noncoding regions can also support tumorigenesis. For instance, mutations or deletions in neighborhood insulators (*i.e.*, genetic boundary elements that block interaction between enhancers and promoters) are found in many types of cancer and are sufficient for activation of oncogenes [10]. Thus, it is well documented that accumulation of genomic alterations in tumors is not only a hallmark, but also a driving force of tumorigenesis in many cancers.

While the consequences of genomic instability in cancers are relatively well-characterized, the causes of genomic instability remain to be clearly defined. It is expected that disruption of processes that function to maintain genome integrity, including the DNA repair system, the chromosome segregation system and multiple cell cycle progression checkpoints, is important in tumorigenesis. Indeed, many types of hereditary cancers are driven by germline mutations in, for instance, DNA repair genes [11,12,13]. However, mutations, for example, in cell cycle checkpoint and DNA repair genes are only present in 31% of sporadic cancers [14,15,16,17]. In the remaining cancers, expression of oncogenes may account for some of the observed genomic instability as a consequence of oncogene-induced replication stress [18].

Recent results suggest that another driver of genomic instability in tumors may be ectopic expression of germ cell genes. Over the last two decades, hundreds of genes that are normally only expressed in the germ cells of the testis have been identified as ectopically expressed in multiple types of human cancer [19,20]. One of the possible reasons for this widespread activation of testis genes in cancer seems to be epigenetic dysregulation resulting in hypomethylation of CpG islands in the regulatory elements of these genes [20,21,22,23,24,25,26]. Many of the encoded proteins are immunogenic when expressed in patient tumors and have been termed cancer/testis antigens [20]. Due to their restricted expression pattern and immunogenic properties, cancer/testis antigens have attracted a lot of attention as potential therapeutic targets. These testes germ cell proteins participate in specialized processes of spermatogenesis, which include sustaining a pool of highly proliferative spermatogonial stem cells and several stages of cellular differentiation into mature sperm. A central element in spermatogenesis is meiosis, where chromosomes duplicate and recombine to exchange genetic material. Recent evidence suggests that ectopic expression of testis germ cell proteins in somatic cells, especially meiosis proteins, can interfere with genomic stability and promote tumorigenesis.

## 2. The Role of Meiotic Proteins in Tumorigenesis

**Figure 1:**
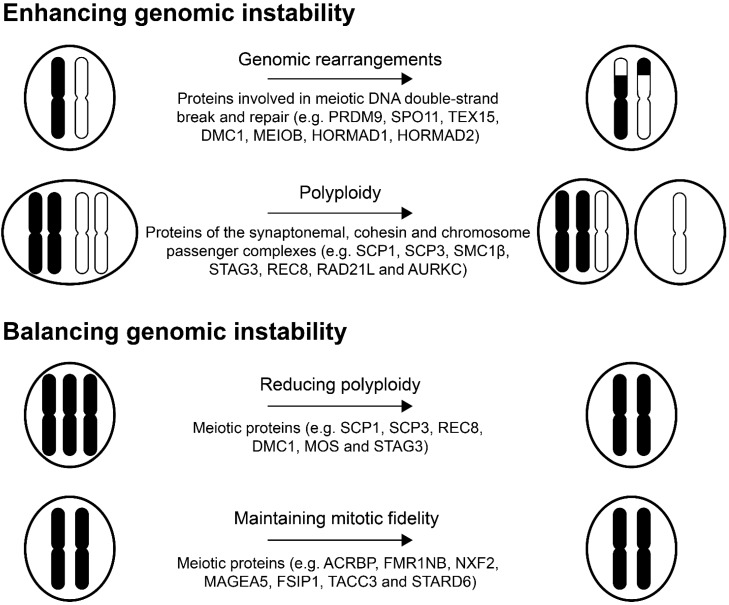
Potential roles of meiotic proteins in genomic instability. Black and white drawings indicate different chromosomes.

Meiosis is a unique feature of germ cells and partly functions to create genetic diversity. Meiosis and mitosis share many features, but while mitosis serves to produce two genetically identical diploid cells, meiosis produces four genetically different haploid gametes. Once germ cells are committed to spermatogenic differentiation, they lose their proliferative capacity and enter the meiosis cycle [27], a process highly controlled by various proteins and complexes. For instance, alignment of the homologous chromosomes is mediated by the synaptonemal complex, while the sister chromatids remain attached by the meiotic chromosome cohesin complex and numerous proteins act in concert to initiate and ensure homologous recombination (reviewed in [28]). Many meiotic proteins are expressed in cancers and may perturb mechanisms maintaining genomic stability (Table 1). For instance, activation of germ cell programs in cancer cells has the capacity to promote chromosomal rearrangements and loss or gain of whole chromosomes (Figure 1).

### 2.1. Proteins Involved in Meiotic Recombination

Meiotic recombination occurs at highly conserved recombination hotspots throughout the genome [95]. A major determinant of these recombination sites is PR Domain Containing 9 (PRDM9) binding, which primes the DNA for double strand break (DSB) and exchange of DNA between chromosomes. This was shown in various mice studies as well as with human PRDM9 [29,30,31]. In mice, Prdm9 mediates trimethylation of lysine 4 at histone 3 (H3K4me3) that, together with other factors, mark chromatin for recombination [96]. This can subsequently recruit Spo11 to initiate a DSB to initiate recombination in mice and yeast (*Saccharomyces cerevisiae*) [29,33,34]. The formation of a DSB is a preceding event of the formation of the synaptonemal complex (discussed below). Interestingly, both *PRDM9* and *SPO11* expression has been demonstrated in multiple cancer cell lines [32,35], and *SPO11* is expressed in melanoma and cervical cancer tumors [36]. In addition, the human *SPO11* gene is found in chromosome 20q13.2–13.3, a region that is amplified in multiple breast cancers and associated with genomic instability, such as aneuploidy, in breast cancer [97,98,99,100]. SPO11 is not entirely restricted to male germ cells [101], thus it may also contribute functions unrelated to DSB formation.

Proteins involved in repairing the DSB generated during meiosis, such as Testis Expressed 15 (TEX15) and Disrupted Meiotic cDNA1 (DMC1), are also expressed in cancer. For instance, *TEX15* and *DMC1* expression are detected in melanoma, sarcoma and various carcinoma tumors [38,40]. In meiosis, Tex15 is responsible for the loading of DNA repair proteins (including Dmc1) onto the DNA following DSB formation, shown in mice [37]. Due to this function, it works downstream of SPO11 and upstream of DMC1 (and other DNA repair proteins). Tex15 has shown limited expression outside the testis (*i.e.*, ovary, brain and uterus) in mice [38,102]. The recombinase DMC1 is a DNA repair protein that associates with single stranded DNA after SPO11-mediated break and end-processing [39,103]. Through this association concurrent with RAD51 (another recombinase, though not meiosis-specific), it searches for homolog sequences between homologous chromosomes and initiates strand invasion wherein the cut DNA strand of a chromatid pair invades a homologous sequence of the other chromatid pair. This, in turn, results in crossover or non-crossover of the flanking regions.

Meiosis Specific with OB Domains (MEIOB) was recently shown to be a DNA binding exonuclease essential for meiotic recombination in synergy with SPATA22, and this protein has been detected in different types of cancer cells [41,42,43]. This is in contrast to *MEIOB* and *SPATA22* expression in normal tissues and lung adenocarcinomas in which expression was found to be mutually exclusive [104]. Importantly, there was a positive correlation between MEIOB and the genome-wide burden of focal copy number aberrations among samples from 10 cancer types with activation of *MEIOB* and *SPATA22* expression. *In vitro* studies further demonstrated that MEIOB enhanced the oncogenic phenotype of lung cancer cells. Moreover, the HORMA Domain Containing 1 (HORMAD1) protein has been linked to genomic instability in cancer. Murine Hormad1 has multiple roles during meiotic recombination: promoting homologue alignment/homologue search by ensuring DSB, facilitating formation of the synaptonemal complex and recruitment of ATR checkpoint kinase activity to unsynapsed chromatin in the meiotic recombination checkpoint [44,45]. In addition, the formation of the synaptonemal complex mediates Hormad1 depletion, which is a requirement for progression beyond the first meiotic prophase [45]. Its diverse expression in cancer includes multiple cancer cell lines from melanoma, breast, lung, ovary and cervical carcinomas (data from the CT Gene Database) [48] and breast, lung, esophageal, endometrial, bladder, colon and gastric carcinoma tumors [46,47,48]. As with MEIOB, HORMAD1 has been associated with genomic instability in the form of allelic-imbalanced copy number aberrations and was found to drive this type of genomic instability by modulating DNA damage repair [47]. The homologous protein HORMA Domain Containing 2 (HORMAD2) also has cancer-testis-associated expression profile [51]. In mice, Hormad2 has a localization pattern in the nucleus similar to Hormad1, suggesting similar functions Hormad1 [49,50].

The proteins discussed above facilitate the exchange of genetic material between chromosomes during meiosis through homologous recombination. A similar mechanism is present in somatic cells to repair DNA damage, and the factors involved in both types of homologous recombination are overlapping. The abnormal expression of one or several meiotic proteins in somatic cells could, in itself or together with endogenously expressed factors, initiate inappropriate recombination events between homologous chromosome structures (Figure 1). This could explain the chromosomal insertions, deletions and translocations frequently observed in cancer cells, although further studies are needed to confirm this.

### 2.2. Proteins Associated with the Synaptonemal Complex

The meiosis-specific synaptonemal complex is a tripartite protein structure that functions to pair homologous chromosomes in the prophase of the first meiotic division. The synaptonemal complex is a multipart structure built up by two lateral elements formed along the axes of the homologous chromosomes and a central element, connected by transverse filaments (several reviews of the synaptonemal complex structure have been published [105,106]). Briefly, the lateral elements consist of Synaptonemal Complex Protein (SCP) 2 and SCP3, while the central elements consists of Synaptonemal Complex Central Element Protein (SYCE) 1-3, Testis Expressed 12 (TEX12) and transverse elements of SCP1. In addition, the lateral elements are associated with meiotic cohesins as well as the above-mentioned HORMAD-proteins. Many of the proteins that constitute the synaptonemal complex are aberrantly expressed in cancers. For example, SCP1 expression has been described in brain, breast, renal cell, gastric, lung, stomach, and pancreatic carcinomas and melanoma (CT Gene Database) [57,58,59,60,61,62,63,107], and SCP3 is expressed in non-small cell lung cancers and leukemia [53,54,108].

The cohesin protein complex is essential for tethering sister chromatids during mitosis, but it also plays a role in meiosis, as depletion of the cohesin components Red8 and Rad21l in mice prevents proper synaptonemal complex function [64,65]. The constitution of cohesin complexes involved in mitosis and meiosis differs, and Structural Maintenance of Chromosomes 1β (Smc1β), Stromal Antigen 3 (Stag3), Rec8 and Rad21l are all subunits unique to meiosis-specific cohesin complexes in mice [66,67]. These proteins are also expressed in different types of cancer [32,35,68,109] and may interfere with the normal function of cohesion during somatic cell mitosis.

Given the role of proteins of the synaptonemal complex and cohesin complexes in pairing of chromosome pairs and sister chromatid tethering in meiosis, it is likely that they interfere with normal chromosome alignment and segregation in mitosis and add to the aneuploidy often observed in cancer (Figure 1). Proteins of the synaptonemal complex may also act in concert with proteins involved in meiotic recombination to produce chromosome rearrangements.

### 2.3. Aurora Kinase C and the Chromosome Passenger Complex

Aurora Kinase C (AURKC) expression is largely limited to cells that undergo meiosis, as shown in both human cells and mouse models [69,70], unlike its closely related family members, Aurora Kinase A (AURKA) and Aurora Kinase B (AURKB), which are expressed in mitotic cells [110]. *AURKC*, like the vast majority of genes described, is a cancer testis antigen [111]. Importantly, AURKC has been shown to be oncogenic as it can transform NIH3T3 cells [110] and is expressed in multiple types of cancer cells [71,72,73,74]. The contribution of AURKC to tumorigenesis remains elusive, but a direct role in promoting genomic instability seems plausible. AURKC is a part of the meiotic chromosomal passenger complex (CPC), important for centrosomal spindle assembly and bipolar orientation of chromosomes during meiosis [112]. Similarly, the CPC of somatic cells, containing AURKB instead of AURKC, is important for bipolar spindle assembly [113]. When AURKC is expressed in somatic cancers, it can interfere with CPC function and spindle assembly checkpoint, likely through promoting AURKB degradation [112]. In line with this, it was demonstrated that overexpression of AURKC in mitotic cells leads to centrosome amplification and multinucleation [110]. As the CPC is important for maintaining chromosome stability during cell division, loss of its function may result in aneuploidy, chromosome imbalances and consequently cancer development [114] (Figure 1).

## 3. Testis Germ Cell Proteins Regulate Polyploidy and Maintain Mitotic Fidelity

In the absence of functional p53, DNA damaging treatments with cytotoxic or genotoxic agents can induce polyploidy and a state of mitotic catastrophe, which is generally lethal to cancer cells. However, some cells will survive long enough to undergo multipolar cell division and, in a fraction of cells, depolyploidization results in continued viability [115,116]. One possible mechanism contributing to this depolyploidization appears to be induced expression of meiotic genes. In a study of radiation-induced mitotic catastrophe in various cancer cell lines, genes specifically associated with meiosis were shown to be upregulated (*i.e.*, *SCP3*, *REC8* and *DMC1*) [40]. These results were confirmed in a similar study based on lymphoma cells wherein additional meiotic genes were found to be upregulated in association with mitotic catastrophe (*i.e.*, *MOS*, *STAG3* and *SCP1*) [117]. The role of REC8 in escape from mitotic catastrophe has been further examined. Mouse Rec8 is important for the segregation of sister chromatids during meiosis and thereby promotes reductional cell division in meiosis [118]. In polyploidy cancer cells, resulting from irradiation-induced mitotic catastrophe, REC8 was associated with centrosomes and spindle poles, further advocating for a role in depolyploidization [119]. Thus, current results suggest that activation of a meiotic program may help tumor cells balance polyploidy.

Interesting, testis germ cell proteins may play yet another role in managing the gross chromosomal abnormalities of cancers cells. Acrosin Binding Protein (ACRBP) was shown to support mitotic fidelity and promote resistance towards paclitaxel in ovarian cancer [120,121]. Further studies demonstrated that ACRBP antagonizes Nuclear Mitotic Apparatus Protein 1 (NUMA1) function in abrogating the mitotic spindle assembly and thereby reinforces bipolar spindle assembly in the presence of paclitaxel. ACRBP exhibits a broad expression pattern in several cancers, including ovarian, lung (NSCLC), breast, bladder, colon and liver, and high expression of ACRBP is associated with a poor clinical outcome in ovarian cancer patients with reduced survival time and faster relapse [81,121]. Additionally, the testis germ cell proteins Fragile X Mental Retardation 1 Neighbor (FMR1NB), Nuclear RNA Export Factor 2 (NXF2), Melanoma Antigen Family A5 (MAGEA5), Fibrous Sheath Interacting Protein 1 (FSIP1), Transforming, Acidic Coiled-Coil Containing Protein 3 (TACC3) and START Domain Containing 6 (STARD6) have a role in mitotic fidelity in cancer cells [82]. Depletion of these proteins from cancer cell lines resulted in the generation of multipolar spindles and induction of micronucleation in response to low doses of paclitaxel. Furthermore, depletion of TACC3 in cells with p53 deletion and/or RASV12 expression increased mitotic transit time and enhanced the frequency of abnormal mitosis. These results clearly demonstrate that cancer cells evolve to depend on testis germ cell proteins for accurate chromosome segregation in the presence mitotic stress (Figure 1).

## 4. Oncogenic Testis Proteins May Promote Replication Stress

Although not directly implicated in genome maintenance, oncogenes may promote genomic instability. There is mounting evidence that oncogenes can induce replication stress in premalignant lesions and cancers, which, in turn, can result in DNA damage and genomic instability [122]. At certain stages of their lifecycle, germ cells are highly proliferative. This is especially true for spermatogonia (*i.e.*, undifferentiated adult male germ cells), which proliferate throughout adult life. It can be speculated that these cells maintain a high level of mitogenic signaling and may express proteins with oncogenic potential. Interestingly, many genes specifically expressed in the spermatogonia are also ectopically activated in multiple types of cancer and, indeed, some of these exhibit oncogenic features [20,123]. For instance, CAGE, MAGE-C, NY-SAR-35, *etc.* promote cancer cell proliferation [110,124,125,126]. Recent results from our group and others suggest that germ cell proteins with oncogenic potential can induce genomic instability in cancer cells in a similar manner to classic oncogenes like those encoding Ras, Myc and Cyclin E [122]. We have demonstrated that knockdown of the *SSX2* gene significantly reduced the proliferation of melanoma cells [125], consistent with another study showing that SSX proteins activate several important mitogenic signaling pathways, such as MAPK and Wnt [127]. We further found that ectopic expression of SSX2 in different cell lines induced DNA damage and, in turn, promoted either a senescence response or genomic instability [125]. In A375 melanoma cells, the underlying course of this phenotype seemed to be replication stress translating into mitotic defects. Whether SSX proteins, or other testis proteins with oncogenic potential, have any role in genomic instability in tumors and contribute to cancer progression remains to be determined.

## 5. Conclusions and Future Perspectives

For cancer cells to exploit genome instability to support tumorigenesis, they must maintain viability and productive mitosis in the presence of gross chromosomal abnormalities. Thus, exploring how cancer cells regulate genomic instability is highly relevant to our understanding of cancer development. Furthermore, many of the currently used treatments induce massive DNA damage and genomic instability to drive cancer cells into apoptosis, and knowledge of how cancer cells cope with these challenges may help us understand drug resistance mechanisms and improve current strategies for cancer treatment. As discussed in this review, current evidence strongly suggests that the activation of testis germ cell genes plays an important role in several aspects of how cancer cells handle genomic instability. During meiosis, germ cell proteins work in concert to carry out DNA breaks, crossover of genetic material and pairing and segregation of chromosomes/chromatids. Testis germ cell proteins are often co-expressed in cancers and it seems plausible that they also functionally interact in cancers to produce genomic instability. Many of these testis germ cell proteins are strictly limited to germ cells in their expression and therefore represent nearly cancer-specific targets. Thus, they are particularly promising as targets for cancer immunotherapy since their germ cell expression does not seem to induce immunological tolerance and allows generation of immune responses in cancer patients [20]. Furthermore, numerous cancer vaccination trials targeting testis germ cell proteins have not shown any effects on spermatogenesis or in any somatic tissue, thus testis germ cell proteins remain ideal candidates for specific targeting of cancer cells. Our current understanding of tumor immunology suggests that immunologic pressure on tumors may result in the evolution of antigen-negative escape variants, highlighting the potential benefits of targeting antigens that, if lost, would reduce the ability of the cancer to further progress [22]. Thus, targeted therapy aimed at proteins supporting basic hallmarks of cancer will likely be highly efficient, and may include testis germ cell proteins regulating genomic instability during cancer development. Such treatment may be combined with DNA damage-inducing agents for synergistic effects. Testis germ cell proteins may therefore be used for development of novel types of highly-specific and effective targeted therapy.

## Figures and Tables

**Table 1:** List of meiotic proteins that potentially regulate genomic instability in cancer.

Protein	Function in Meiosis	Species of Functional Studies	Expression in Cancer
PRDM9	Meiotic recombination hotspot activator [29,30,31]	Mice and human protein	Embryonal carcinoma, astrocytoma, colon, prostate, breast, ovary, melanoma and leukemia cancer cell lines [32]
SPO11	Meiosis-specific nuclease [29,33,34]	Mice and yeast	Melanoma and lung cancer cell lines, and melanoma and cervical cancer tissue [35,36]
TEX15	Mediates loading of DSB repair proteins onto DNA (at DSB sites) [37]	Mice	Bladder carcinomas, cutaneous melanoma, esophageal carcinomas, head and neck carcinomas, lung carcinoma, neuroblastomas, prostate tumors, renal tumors and sarcomas [38]
DMC1	Recombinase/DNA repair protein [39]	Human protein	Cervical cancer tissue [40]
MEIOB	3′ to 5′ exonuclease [41,42,43]	Mice and human cell lines	Liver, leukemia and lung cancer cell lines [41]
HORMAD1	Mediates homologous recombination, synaptonemal complex formation and recruitment of ATR kinase activity to unsynapsed chromatin [44,45]	Mice	Gastric [46], breast (including triple-negative breast cancer), non-small cell lung cancer (NSCLC), esophageal, endometrical, bladder and colon cancer tissue [47,48], and breast, ovarian, melanoma, cervical, NSCLC and small lung cancer cell lines (CT Gene Database) [48]
HORMAD2	Proposed function: Similar to HORMAD1 [49,50]	Mice	Lung cancer tissue [51]
SCP-3/SYCP3	Synaptonemal complex protein [52]	Mice	NSCLC [53] and cervical cancer tissue [54]
SYCE1	Synaptonemal complex protein [55]	Mice	Breast cancer, melanoma and leukemia cancer cell lines (CT Gene Database)
SCP-1/SYCP1	Synaptonemal complex protein [56]	Human cell line	Breast, melanoma, brain (glioma, glioblastoma, schwanoma, medulloblastoma, meningioma, astrocytoma, oligoastrocytoma and pilocytic astrocytoma), gastric, lung (including NSCLC), renal cell, stomach [57,58,59,60,61] and pancreatic cancer tissue [62], and ovarian (CT Gene Database), ATLL [63] and pancreatic [62] cancer cell lines
REC8	Component of meiosis-specific cohesin complex [64,65,66,67]	Mice	Melanoma cell lines [35,68]
RAD21L	Component of meiosis-specific cohesin complex [64,65,66,67]	Mice	Colon, breast, ovarian, embryonal carcinoma, cervix and leukemia cancer cell lines [32]
SMC1β	Component of meiosis-specific cohesin complex [64,65,66,67]	Mice	Breast, leukemia and embryonal carcinoma cancer cell lines [32]
STAG3	Component of meiosis-specific cohesin complex [64,65,66,67]	Mice	Various cancers (reported in Oncomine, September 2012)
AURKC	Component of the of the meiotic chromosomal passenger complex [69,70]	Mice and human cell lines	Breast, cervical, liver, prostate, thyroid carcinoma cancer cells lines [71,72,73], and colorectal and thyroid cancer tissue [71,74]
MOS	Regulates oocyte maturation [75]	Xenopus	Ovarian cancer tissue [76] and neuroblastoma and cervical carcinoma-derived cell lines [77]
ACRBP	Role in spermatogenesis and sperm capacitation [78,79]	Porcine	Ovarian cancer cell line and cancer tissue [80], bladder, breast, lung, liver and colon cancer tissue [81], and sarcoma, prostate, multiple myeloma, chronic myeloid leukemia, lung and ovarian cancer cell lines (CT Gene Database)
FMR1NB	Role in regulating microtubule nucleation and/or anchoring events in the mitotic spindle (suggested role from CT Gene Database, based on [82])	Human cell lines	Melanoma, sarcoma, lung, breast, bladder, esophageal and ovarian cancer tissue [83], and sarcoma, multiple myeloma, chronic myeloid leukemia, choriocarcinoma, lung and breast cancer cell lines (CT Gene Database)
NXF2	Nuclear RNA export factor, important for regulation of meiosis and maintenance of spermatogonial stem cells [84]	Mice	Melanoma, sarcoma, prostate, multiple myeloma, chronic myeloid leukemia, choriocarcinoma, lung, ovarian and colon cancer cell lines (CT Gene Database), and bladder, colorectal carcinoma, lung, melanoma, esophageal, head and neck, neuroblastoma, prostate, sarcoma and thyroid cancer tissue [38]
MAGEA5			Breast [85], lung, head and neck [86], lung adenocarcinoma and squamous cell cancer tissue [87], and melanoma and thyroid carcinoma cell lines [88]
FSIP1	Component of the fibrous sheath structure, unique for spermatogenic cells [89]	Mice and yeast	Breast cancer tissue [90,91]
TACC3	Mitosis: Plays a role in spindle stability and kinetochore-microtubule interactions [92]	Human cell lines	Breast, lung, colon and liver cancer tissue [93]
STARD6	Involved in transport of lipids [94]	Mice	
